# IL-6 Promoter Hypomethylation Acts As a Diagnostic Biomarker in Hepatitis B Virus-Associated Hepatocellular Carcinoma

**DOI:** 10.3389/fonc.2022.746643

**Published:** 2022-03-11

**Authors:** Jie-Ru Yang, Ju Wang, Hai-Ming Li, Shuai Gao, Yu-Chen Fan, Kai Wang

**Affiliations:** ^1^ Department of Hepatology, Qilu Hospital of Shandong University, Jinan, China; ^2^ Institute of Hepatology, Shandong University, Jinan, China

**Keywords:** hepatocellular carcinoma, methylight, biomarkers, promoter methylation, IL-6

## Abstract

**Background:**

New biomarkers are needed to detect hepatocellular carcinoma at an earlier stage and to individualize treatment strategies. IL-6 has been proven to be associated with liver cancer in numerous studies.

**Aim:**

To evaluate the value of the IL-6 promoter methylation level as a noninvasive biomarker for the diagnosis of liver cancer.

**Methods:**

A retrospective analysis of 165 patients with HBV-associated hepatocellular carcinoma (HCC), 198 patients with chronic hepatitis B (CHB) and 31 healthy controls were involved. The methylight was detected the methylation level of the IL-6 promoter in peripheral blood mononuclear cells (PBMCs), clinical and laboratory parameters were obtained.

**Results:**

IL-6 promoter methylation levels were significantly lower in patients with HCC (median 53.59%, interquartile range 52.01–54.75%) than in those with CHB (median 56.05%, interquartile range 54.65–57.67%; P<0.001). The level of IL-6 mRNA in patients with HCC (median 0.371, interquartile range 0.173-0.671) was significantly higher than that in patients with CHB (median 0.203, interquartile range 0.108-0.354; P<0.001) and HCs (median 0.189, interquartile range 0.140-0.262; P=0.001). Meanwhile, the PMR value of IL-6 was notably negatively correlated with the mRNA expression level (Spearman’s R=-0.201, P<0.001). The IL-6 PMR value of HCC patients in age (Spearman’s R=0.193, P=0.026) and TBIL (Spearman’s R=0.186, P=0.032) were very weak correlated. At the same time, the level of IL-6 promoter methylation was also an independent factor in the development of liver cancer. When the IL-6 promoter methylation level was used to diagnose HCC, its detective value was superior to AFP [area under the receiver operating characteristic curve (AUC) 0.773 *vs*. 0.686, P=0.027], And the combined use of AFP and IL-6 methylation level can improve the area under the receiver operating characteristic curve (p=0.011).

**Conclusion:**

IL-6 promoter hypomethylation is present in hepatocellular carcinoma, and it may be used as a noninvasive biomarker to detect early liver cancer.

## Introduction

Hepatocellular carcinoma (HCC) accounts for approximately 90% of liver cancer, with an incidence rate of 4.7% of all tumors, the sixth most common tumor in the world, and a fatality rate of 8.3% of all tumors, becoming the leading cause of cancer-related deaths. Meanwhile, it is the third leading cause, the most common form of liver cancer, and the leading cause of liver cancer diagnosis and death (1, 2). In Western countries, metabolic syndrome or diabetes-related nonalcoholic steatohepatitis are more common risk factors, but in China, hepatitis B virus (HBV) and hepatitis C virus infection are the main risk factors for the development of HCC (3, 4). Currently, the diagnosis methods of HCC mostly include alpha-fetoprotein (AFP), ultrasound, computed tomography scan and magnetic resonance imaging (5, 6). However, the sensitivity of AFP is limited and the efficiency of imaging diagnosis is related to the level of imaging doctors (7). Because of the difficulty of early diagnosis, a large number of liver cancer patients are easily missed diagnosis and diagnosed at an advanced stage, especially in China (8). In addition, advanced liver cancer is characterized by a high degree of malignancy, invasion and low sensitivity to chemotherapeutic drugs which leads to difficulty in treatment and poor prognosis (9, 10). Therefore, there is an urgent need for a better noninvasive and sensitive biomarker for detection.

The occurrence of tumors is related to many factors. Abnormal DNA methylation of CpG islands has been widely observed in the occurrence of various tumors, such as colorectal cancer (11, 12), thyroid cancer, lung, breast, and prostate cancers (13). Recent studies have found that abnormal DNA methylation is associated with many human diseases, and the DNA methylation status of free cells is similar to that of primary tumor tissues (12, 14–16), and can be used as a biomarker for disease detection and prognosis prediction. Methylight, a sensitive real-time PCR technology, is highly specific and sensitive in methylation detection compared to other methods for detecting methylation levels and is suitable for detecting low-frequency DNA methylation biomarkers (15, 17).

Interleukin-6 (IL-6) is a four-helix cytokine composed of 184 amino acids (18). Its synthesis and secretion are influenced by inflammatory conditions (18). In a variety of cancers, IL-6/JAK/STAT3 is overactivated, which promotes tumor cell proliferation, survival, invasiveness and metastasis and inhibits the antitumor immune response, which is related to the poor prognosis of tumors (19). Relevant studies have now shown that the IL-6 promoter inhibits DNA methylation in prehypertensive youngsters (20) and that CpG methylation of the IL-6 gene can be used as a surrogate biomarker for the diagnosis of IBD in children (21). At the same time, IL-6 is involved in the occurrence of HBV-associated HCC by activating the STAT3 pathway (22). It is possible that the hypomethylation of the IL-6 promoter also occurs in HCC patients, which can be used as a noninvasive candidate biomarker with potential clinical value.

In this study, we used MethyLight to detect the methylation status of the IL-6 promoter in patients with HBV-associated HCC, chronic hepatitis B (CHB) and healthy controls (HCs) as well as analyze the correlation between the IL-6 promoter methylation level and other clinicopathological characteristics. We then attempted to evaluate the potential clinical value of the IL-6 methylation level as a noninvasive biomarker for the diagnosis of HCC.

## Materials and Methods

### Patients and Controls

In our study, 165 patients with HCC, 198 patients with chronic hepatitis B (CHB) and 31 healthy controls (HCs) were retrospectively enrolled from July 2017 to January 2020 at the Department of Hepatology, Qilu Hospital of Shandong University. All patients with HCC were infected with the hepatitis B virus. The HCC patients were enrolled according to the diagnostic criteria delineated in the 2018 updated American Association for the Study of Liver Diseases (AASLD) Practice Guidelines for the Management of HCC (6). The diagnosis of CHB was made according to the guidelines for the 2018 updated American Association for the Study of Liver Diseases (AASLD) (23). Subjects with the following situations were excluded: coinfection with hepatitis A virus, hepatitis C virus, hepatitis D virus, or hepatitis E virus; pregnancy; coexistence with other liver diseases, such as autoimmune hepatitis, alcoholic hepatitis, or drug hepatitis; metabolic disorders; and human immune deficiency virus (HIV) infection; HBsAg negative; coexistence with other tumors; incomplete data; and withdrawal.

Prior to sample collection, informed consent was obtained from every participant and the study was approved by the local Ethical Committee of Qilu Hospital of Shandong University, with the guidelines of the 1975 Declaration of Helsinki.

### Plasma Collection and Peripheral Blood Mononuclear Cells (PBMCs) Isolation

Venous peripheral blood of five milliliters was drawn from participants on the first day of diagnosis, using EDTA as an anticoagulant agent. PBMCs were isolated by gradient centrifugation from the blood *via* Ficoll-Plaque Plus (GE Healthcare, Uppsala, Sweden). Then, PBMCs from the interface were collected and washed three times with phosphate-buffered saline and stored at –20°C until use.

### Sodium Bisulfite Modification

DNA bisulpfate modification was performed by an EZ DNA Methylation-Gold Kit (Zymo Research, Orange, CA, USA) according to the manufacturer’s instructions. A final volume of 20 μl modified DNA was obtained and was either used immediately as a template for MethyLight or stored at -20°C until use.

### TaqMan Probe-Based Quantitative Methylation Specific Polymerase Chain Reaction (MethyLight)

The methylation level of the IL-6 promoter was detected using MethyLight analysis, as used in previous research (17). Briefly, two sets of primers and probes designed specifically for bisulfite-converted DNA were used: a methylated set for the IL-6 gene and a reference set for the ACTB gene to normalize for input DNA. ACTB gene specific primers and probes were designed as previously described (24). The gene sequence of the IL-6 promoter was obtained from the website http://genome.ucsc.edu/. The 6 CpG motifs of the IL-6 gene promoter from The region –1083 bp to -952 bp (25). The forward and reverse primers, and probes were designed using the oligo7 software tool (OLIGO 1267 Vondelpark Colorado Springs, CO 80907, USA). The sequences are listed in **Table 1**.

**Table 1:** Sequences of the primers and probes used.

Gene	Forward primer sequence (5’-3’)	Reverse primer sequence (5’-3’)	Probe oligo sequence
Methylight			
IL-6	TTATATTATATAGACGGATTATAGTGTACGG	TTTTAATACTCTCCTATCTTAAACAACGTA	AAAACGAAACCACTACTCCCAACTCCGCAA
ACTB	TGGTGATGGAGGAGGTTTAGTAAGT	AACCAATAAAACCTACTCCTCCCTTAAA	ACCACCACCCAACACACAATAACAAACACA
RT–qPCR			
IL-6	ATGCAATAACCACCCCTGAC	GAGGTGCCCATGCTACATTT	
β-actin	ATGGGTCAGAAGGATTCCTATGTG	CTTCATGAGGTAGTCAGTCAGGTC	

The total volume of the MethyLight assays was 10 μL, containing 2 μL nuclease-free water, 5 μL MethyLight Master Mix consisting of HotStarTaq Plus DNA Polymerase, EpiTect Probe PCR Buffer and dNTP mix (dATP, dCTP, dGTP, dTTP), 0.4 μL forward and reverse primers, 0.2 μL Taqman probe and 2 μL of bisulfite conversion DNA composition. MethyLight was performed on Agilent Technologies Stratagene Mx3005P instrument (Stratagene, La Jolla, CA) under the following conditions. 95°C for 15 min, followed by 50 cycles of 95°C for 15 s and 60°C for 1 min. Human control DNA (QIAGEN, Hilden, Germany) transformed with SSSI methylase and bisulfite *in vitro* was used as a reference for methylation. The methylight data are expressed as a percentage of the methylation reference value (PMR). Each sample was carried out in triplicate. Each plate included at least three control wells without the template, as well as negative and positive controls.

PMR = 100% × 2 exp−[Delta Ct (target gene in sample−control gene in sample) −(Delta Ct 100% methylated target in reference sample−control gene in reference sample)] (26)

### Quantitative Real-Time Polymerase Chain Reaction (RT–PCR)

Total RNA of PBMCs was extracted by TRIzol (Invitrogen, Carlsbad, CA, USA). The RNA concentration was determined by an Eppendorf Biophotometer (Brinkmann Instruments, Westbury, NY). RNA was then converted into cDNA *via* a first-strand cDNA synthesis kit (Fermentas, Vilnius, Lithuania). The expression of IL-6 mRNA was detected by real-time PCR, which was performed on a Lightcycler 480 (Roche Diagnostic, Mannheim, Germany) with SYBR Green (Toyobo, Osaka, Japan). β-Actin was used as the endogenous control. Amplification was performed in a total volume of 20 μL, which contained 0.5 mM of cDNA, 0.5 mM of each primer and 10×SYBR Green. The primers were described in **Table 1**. The reaction of PCR was performed as follows: the initial step was 95°C for 30 s, followed by 50 cycles of 95°C for 5 s, 55°C for 30 s, and a final step of 72°C for 30 s. Comparative real-time RT–PCR assays were performed for each sample in triplicate. The IL-6 mRNA levels were calculated using the comparative2(–ΔΔCt) method.

### Clinical Data Collection

The following markers were measured by standard methods in the Laboratory of Shandong University Qilu Hospital. Serum biochemical markers (COBAS Integra 800; Roche Diagnostics) included aspartate aminotransferase (AST), alanine aminotransferase (ALT), total bilirubin (TBIL), serum alpha-fetoprotein (AFP), albumin (ALB), HBV-DNA load and HBeAg. Hemostatic markers (ACL TOP 700; Instrument Laboratory, Lexington, MA, USA) included the prothrombin time-international normalized ratio (PT-INR) and prothrombin time activity (PTA). The Child–Pugh classification was used to evaluate liver function from the original indicators at the time of admission. All imaging results, including computed tomography and magnetic resonance imaging data, were evaluated by a radiologist who did not know the characteristics of the patient. All tissue specimens were evaluated by a pathologist who did not know the characteristics of the patient. Histopathological data, including tumor size and vascular infiltration, were collected from patient records. In addition, HCC patients were divided into two subgroups according to Barcelona (BCLC) staging. Stages 0, A and B are the early stages, and stages C and D are the advanced stages (6, 27).

### Statistical Analysis

All statistical analyses of the data were performed using the IBM SPSS version 26.0 (SPSS Inc., Chicago, IL, USA). The Kolmogorov–Smirnov test was performed to determine whether the data were from a normal distribution population. Quantitative variables are expressed as the median (centile 25; centile 75). Categorical variables were expressed as numbers (%). Quantitative variables were compared using the Mann–Whitney U test and the Kruskal–Wallis H test. Spearman’s test was applied to determine the relationship between the IL-6 methylation level and quantitative clinical data. The diagnostic value of the IL-6 methylation level and AFP score in the diagnosis of HCC patients was assessed by the area under the receiver operating characteristic curve (AUC). At the same time, a model based on binary logistic regression was established to evaluate the value of the combined diagnosis of IL-6 methylation level and AFP. From the receiver operating characteristic (ROC) curve coordinates, the optimal cutoff point associated with the maximum Youden index was determined. Sensitivity, specificity, positive predictive value (PPV) and negative predictive value (NPV) were used to assess the diagnostic accuracy. Multiple logistic regression analysis was used to determine independent risk factors for liver cancer. All statistical analyses were two-sided and P-value < 0.05 was considered statistically significant.

## Results

### General Characteristics of Subjects

**Figure 1:**
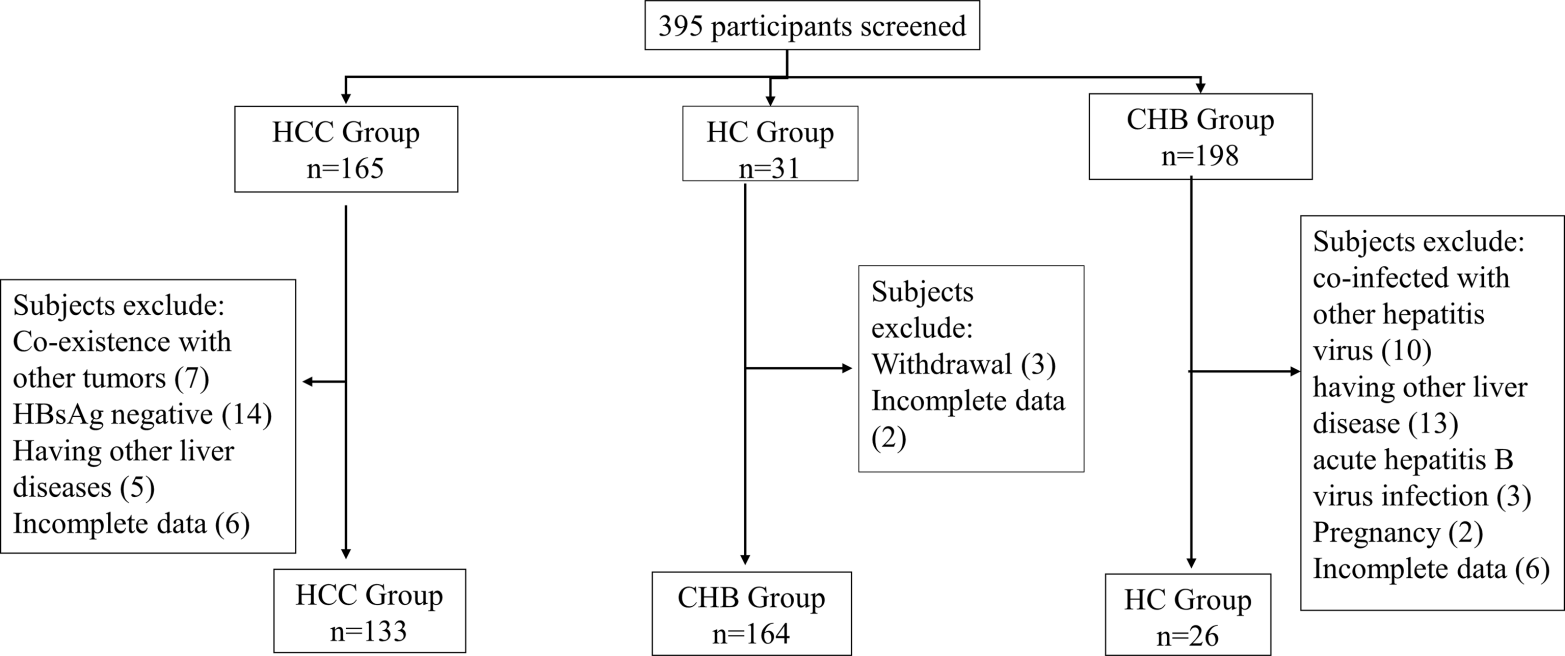
Patient selection process.

The selection process for the enrolled subjects is shown in **Figure 1**. A total of 395 subjects were initially screened and 323 subjects were admitted to this study, including 133 patients with HCC, 164 patients with chronic hepatitis B (CHB) and 26 healthy controls (HCs). The baseline characteristics of the enrolled subjects are shown in **Table 2**.

**Table 2:** Baseline characteristics of the enrolled participants.

Variable	HCC group (n = 133)	CHB group (n = 164)	HC group (n = 26)
Age (years)	56 (51-62)	49 (40-56)	48.5 (35.75-55)
Male, n (%)	106 (79.7)	119 (72.6)	11 (42.3)
ALT (U/L)	38 (22.00-72.50)	36.85 (25.00-90.75)	24.50 (15.00-32.00)
AST (U/L)	44 (31.00-107.00)	47.00 (32.00-87.00)	31.50 (22.50-38.25)
TBIL (μmol/L)	22.10 (14.30-39.75)	25.40 (14.55-57.45)	9.00 (8.00-15.00)
ALB (g/L)	39.00 (34.05-43.70)	36.10 (31.40-41.38)	49.50 (46.75-52.25)
PT-INR	1.19 (1.11-1.29)	1.28 (1.14-1.58)	NA
PTA (%)	77 (67-84)	68.5 (53-82.5)	NA
AFP (ng/ml)	74.70 (4.62-800.00)	6.94 (2.68-33.52)	NA
HBV-DNA (+), n (%)	72 (54.1)	122 (74.4)	NA
HBeAg (+), n (%)	72 (54.1)	102 (62.2)	NA
Encephalopathy (%)	10 (7.5)	17 (10.3)	NA
Ascites (%)	47 (35.3)	21 (12.8)	NA

Quantitative variables are expressed as the median (centile 25; centile 75). Categorical variables are expressed as numbers (%). HCC, hepatocellular carcinoma; CHB, chronic hepatitis B; HC, healthy control; HBV, hepatitis B virus; AFP, alpha-fetoprotein; AST, aspartate aminotransferase; ALT, alanine aminotransferase; ALB, albumin; TBIL, total bilirubin; INR, international normalized ratio; PTA, prothrombin time activity; HBeAg, hepatitis B e surface antigen; NA, not available.

### Methylation Status of the IL-6 Promoter in Different Groups

**Figure 2:**
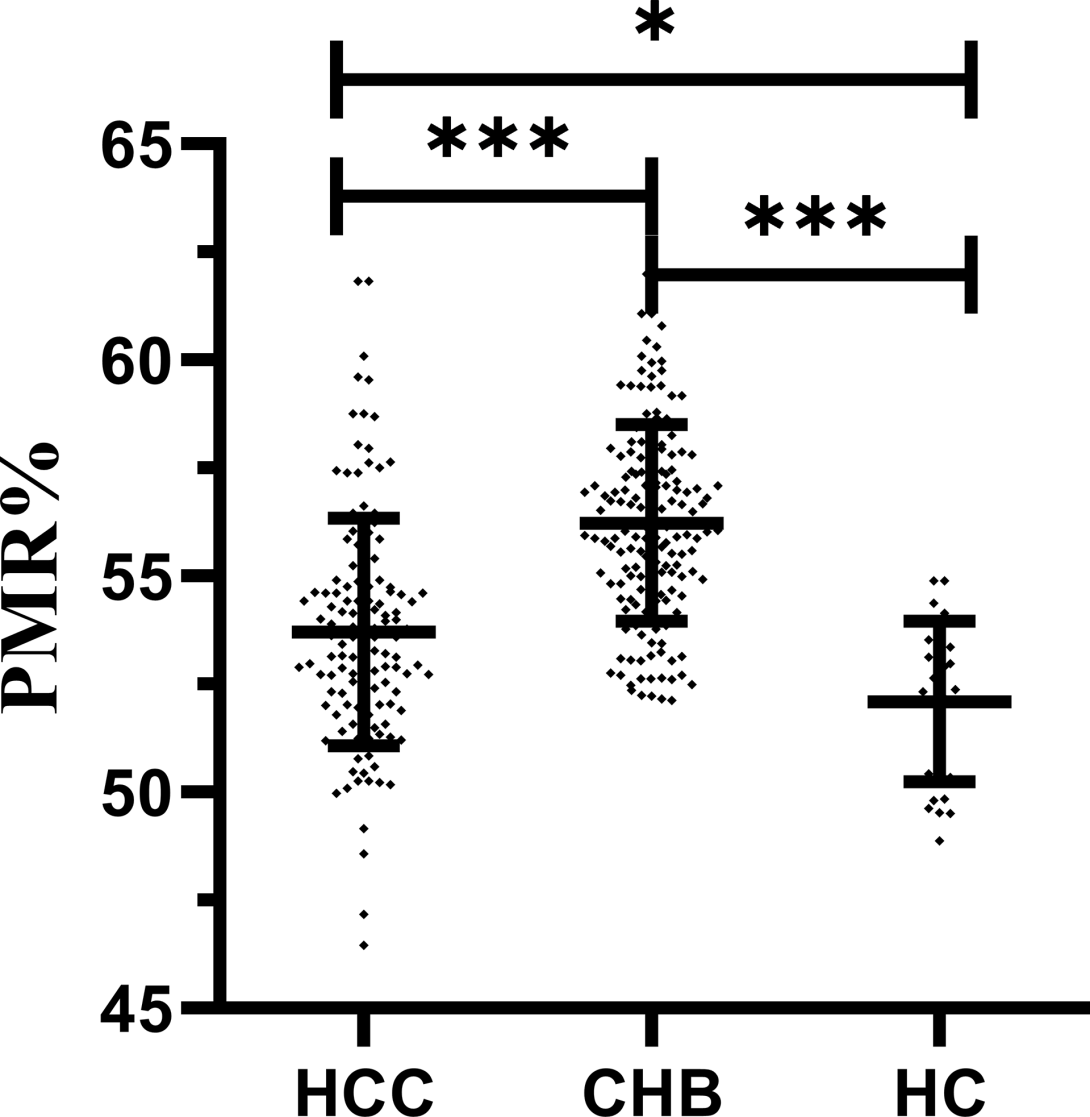
IL-6 promoter methylation level in patients with HBV-associated HCC, CHB and HCs. The IL-6 methylation level was significantly lower in patients with HBV-associated HCC than in those with CHB (p < 0.001) but higher than that in HCs (p = 0.026). Meanwhile, methylation levels of the IL-6 promoter were significantly higher in CHB patients than in healthy controls (P < 0.001) ***p < 0.001, *p < 0.05.

The methylation level of the IL-6 promoter using the PMR values of participants in different groups is shown in **Figure 2**. The IL-6 promoter methylation level was significantly lower in patients with HBV-associated HCC (median 53.59%, interquartile range 52.01–54.75%) than those with CHB (median 56.05%, interquartile range 54.65–57.67%; P<0.001) but higher than that in HCs (median 52.51%, interquartile range 50.21–53.56%; P=0.026). Meanwhile, the methylation level of the IL-6 promoter was notably higher in CHB patients than in healthy controls (P<0.001).

### IL-6 mRNA Levels in Different Groups

**Figure 3:**
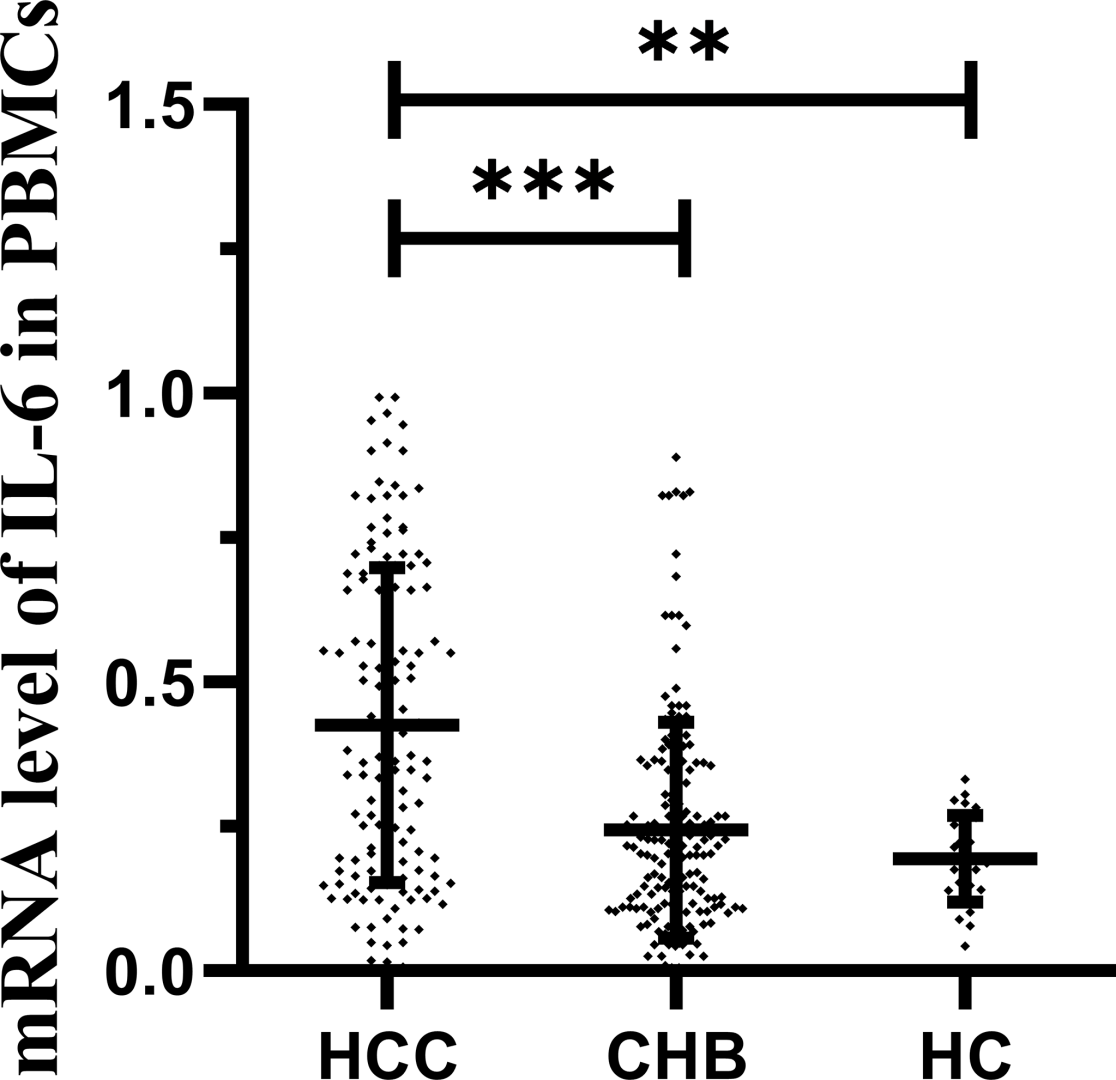
IL-6 mRNA levels in patients with HBV-associated HCC, CHB and HCs. IL-6 mRNA level was significantly higher in the HCC group than in the CHB (p < 0.001) and HCs (P = 0.001). ***p < 0.001, **p < 0.01.

**Figure 4:**
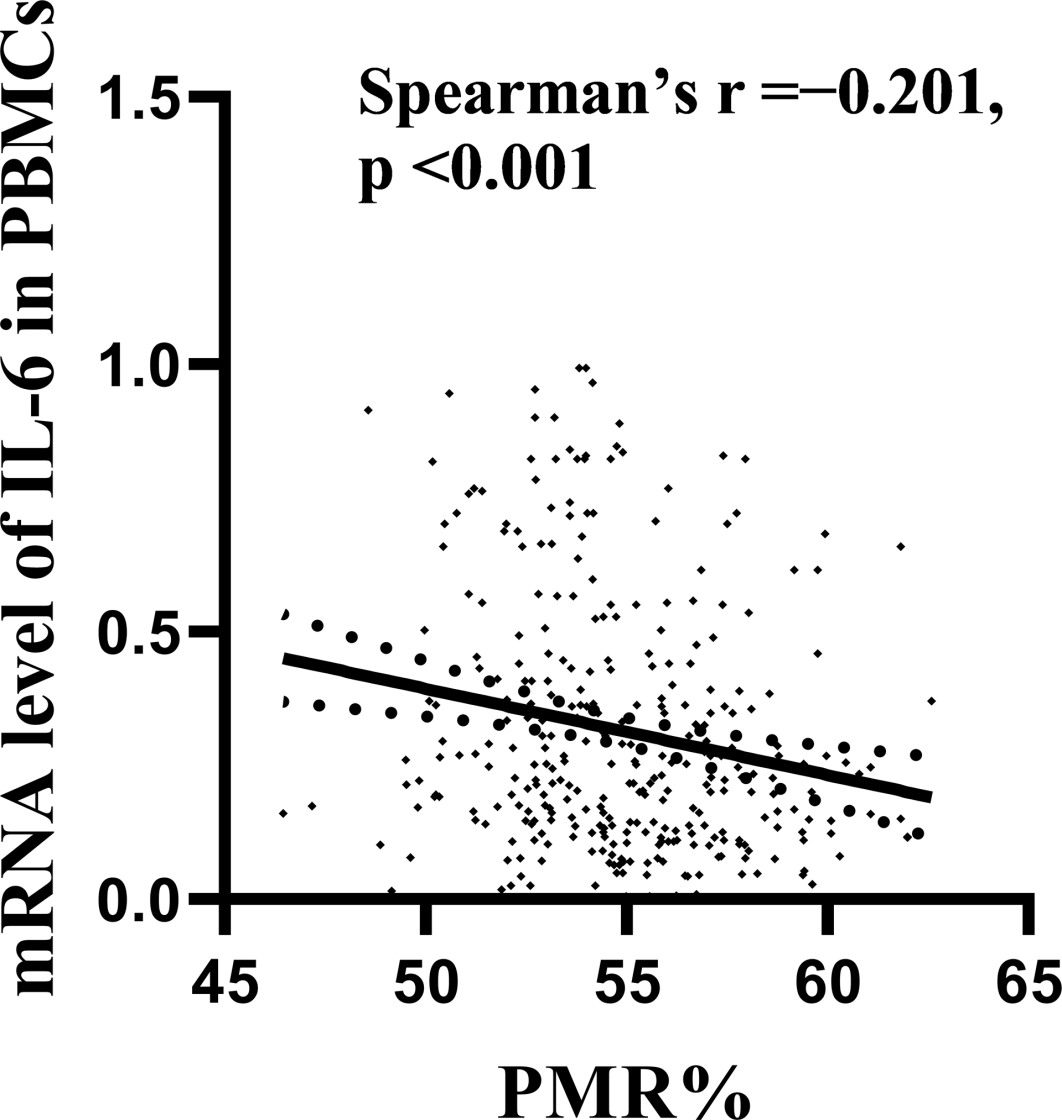
Relationships between IL-6 promoter methylation levels and mRNA levels in PBMCs. A significant correlation was observed between the PMR value of the IL-6 promoter and the mRNA level in PBMCs (Spearman’s r=-0.201, P < 0.001).

The expression level of IL-6 mRNA in PBMCs was detected by RT–PCR. The results are shown in **Figure 3**. An increasing trend of IL-6 expression corresponding to disease progression was observed. The level of IL-6 mRNA in patients with HCC (median 0.371, interquartile range 0.173-0.671) was remarkably higher than that in patients with CHB (median 0.203, interquartile range 0.108-0.354; P<0.001) and HCs (median 0.189, interquartile range 0.140-0.262; P=0.001). There was no significant difference between the CHB and HC groups. This is consistent with our previous study (28, 29). To further analyze the relationship between the IL-6 methylation level and mRNA expression level, we used Spearman rank correlation analysis and found that the PMR value of IL-6 was weakly negatively correlated with mRNA expression levels (Spearman’s R=-0.201, P<0.001) **Figure 4**.

### Associations Between IL-6 Promoter Methylation Levels and Clinicopathological Features in HCC

**Figure 5:**
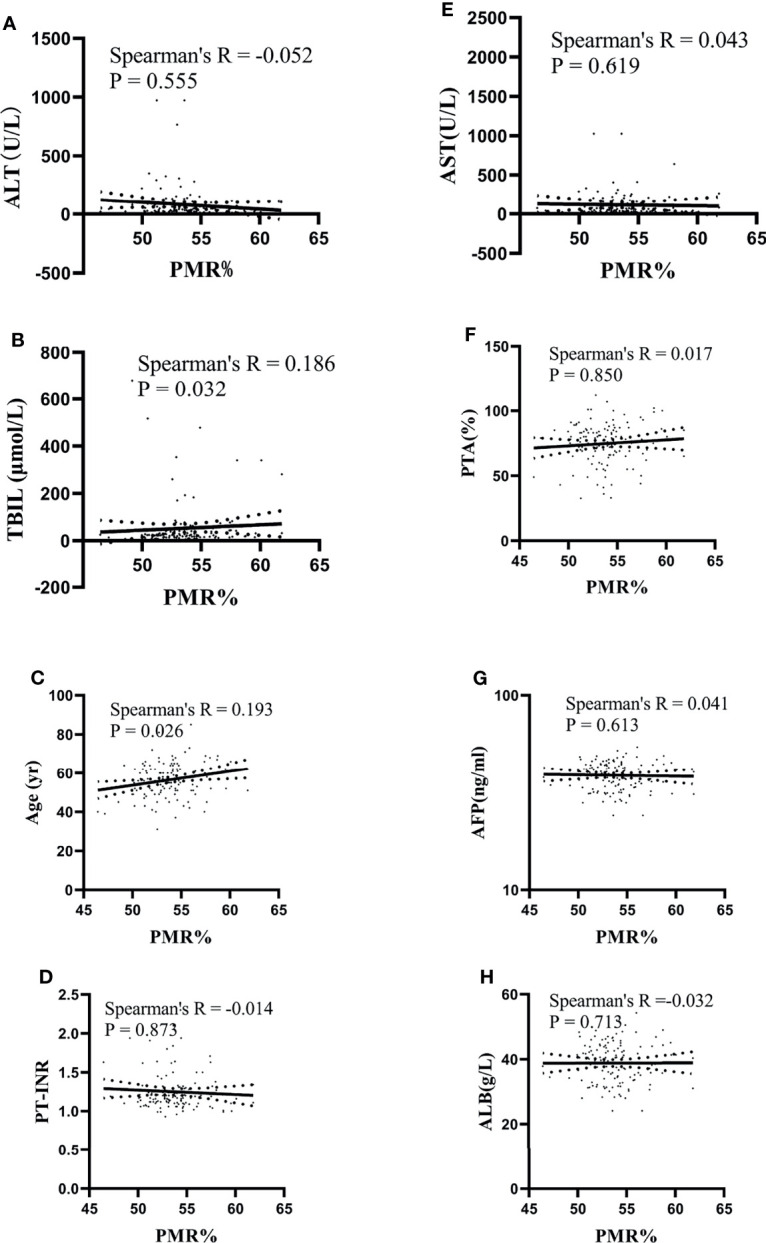
Relationships between the IL-6 promoter methylation level and quantitative clinical data in the HCC group. **(A)** The PMR value of the IL-6 promoter was not significantly correlated with ALT in patients with HCC (Spearman’s R = -0.052, P = 0.555). **(B)** The PMR value of the IL-6 promoter was significantly correlated with TBIL in patients with HCC (Spearman’s R = 0.186, P = 0.032). **(C)** The PMR value of the IL-6 promoter was significantly correlated with age in patients with HCC (Spearman’s R = 0.193, P = 0.026). **(D)** The PMR value of the IL-6 promoter was not significantly correlated with PT-INR in patients with HCC (Spearman’s R = -0.014, P = 0.873). **(E)** The PMR value of the IL-6 promoter was not significantly correlated with AST in patients with HCC (Spearman’s R = 0.043, P = 0.619). **(F)** The PMR value of the IL-6 promoter was not significantly correlated with PTA in patients with HCC (Spearman’s R = 0.017, P = 0.850). **(G)** The PMR value of the IL-6 promoter was not significantly correlated with AFP in patients with HCC (Spearman’s R = 0.041, P = 0.613). **(H)** The PMR value of the IL-6 promoter was not significantly correlated with ALB in patients with HCC (Spearman’s R =-0.032, P = 0.713).

In HBV-associated HCC patients, the correlation between IL-6 promoter methylation levels and clinical parameters was analyzed. As shown in **Table 3**, the level of IL-6 promoter methylation was significantly higher in males (median 53.70%, interquartile range 52.03-55.00%) than in females (median 52.07-54.16%, P=0.028), in HBV-DNA positive patients (median 53.96%, interquartile range 52.33-55.41%) than in HBV-DNA negative patients (53.18% of median, interquartile range 51.74-54.61%, P=0.044), and in patients more than 50 years old (median 53.73%, interquartile range 52.33-54.82%, P=0.040) than in patients less than 50 years old (median 52.54%, interquartile range 51.23-54.57%). Meanwhile, the IL-6 PMR value in the tumors whose diameter was more than the 3 cm (median 53.81%, interquartile range 51.85-55.97%) was significantly higher than that in tumors whose diameter was less than or equal to 3 cm (53.42%, interquartile range 52.01-54.43%, P=0.032). The IL-6 promoter methylation level showed no significant difference in HBeAg (P=0.314), AFP (Ng/mL) (p=0.532), primary tumor number (P=0.801), vascular invasion (P=0.658), CTP staging (P=0.308), BCLC staging (P=0.389), ascites (P=0.212), encephalopathy (P=0.082). Continue to test the relationship using Spearman rank correlation test between IL-6 promoter methylation and age, AFP, AST, ALT, TBIL, PTA%. The IL-6 PMR value of HCC patients in age (Spearman’s R=0.193, P=0.026) and TBIL (Spearman’s R=0.186, P=0.032) were very weak correlation. However, the IL-6 promoter methylation level and AFP (Spearman’s R=0.041, P=0.613), AST (Spearman’s R=0.043, P=0.619), ALT (Spearman’s R=-0.052, P=0.555), PTA% (Spearman’s R=0.017, P=0.850), PT-INR (Spearman’s R=-0.014, P=0.873), PTA% (Spearman’s R=0.017, P=0.850), and ALB (Spearman’s R=-0.032, P=0.713) were not correlated. (**Figure 5**)

**Table 3:** Associations between IL-6 promoter methylation levels and clinicopathological features in HCC.

Parameters	Total number	PMR (%)	P value
Gender			0.028a*
Male	106	53.70 (52.03-55.00)	
Female	27	52.72 (51.07-54.16)	
Age (year)			0.040a*
<=50	28	52.54 (51.23-54.57)	
>50	105	53.73 (52.33-54.82)	
HBeAg			0.314^a^
Negative	70	53.18 (51.74-54.61)	
Positive	63	53.96 (52.33-55.41)	
HBV-DNA			0. 044^a*^
Negative	63	53.67 (52.40-55.85)	
Positive	70	53.24 (51.53-54.58)	
AFP (ng/ml)			0.532^a^
<=20	53	53.41 (52.03-54.60)	
>20	80	53.68 (51.82-55.25)	
Primary tumor number			0.801^a^
single	94	53.58 (52.02-54.75)	
multiple	39	53.62 (51.34-54.91)	
Tumor size			0.032^a*^
<=3 cm	73	53.42 (52.01-54.43)	
>3 cm	60	53.81 (51.85-55.97)	
Vascular invasion			0.658^a^
Negative	73	53.83 (52.15-54.70)	
Positive	60	53.13 (51.97-54.84)	
CTP staging			0.308^b^
A	61	53.72 (51.87-55.06)	
B	36	53.52 (52.48-54.75)	
C	36	54.40 (52.00-54.67)	
BCLC staging			0.389^a^
0/1/2	83	53.63 (52.02-54.76)	
3/4	50	53.50 (51.72-54.77)	
Ascites			0.212^a^
No	86	8.12 (4.50-20.97)	
Yes	47	6.95 (1.88-22.31)	
Encephalopathy			0.082^a^
No	124	7.58 (2.72-18.92)	
Yes	9	4.77 (2.99-9.41)	

CTP, Child–Turcotte–Pugh; BCLC, Barcelona-Clinical-Liver-Cancer.

*p<0.05.

**p<0.01.

***p<0.001.

^a^ann–Whitney U test.

^b^Kruskal–Wallis H test.

### Diagnostic Value of the IL-6 Promoter Methylation Level

**Figure 6:**
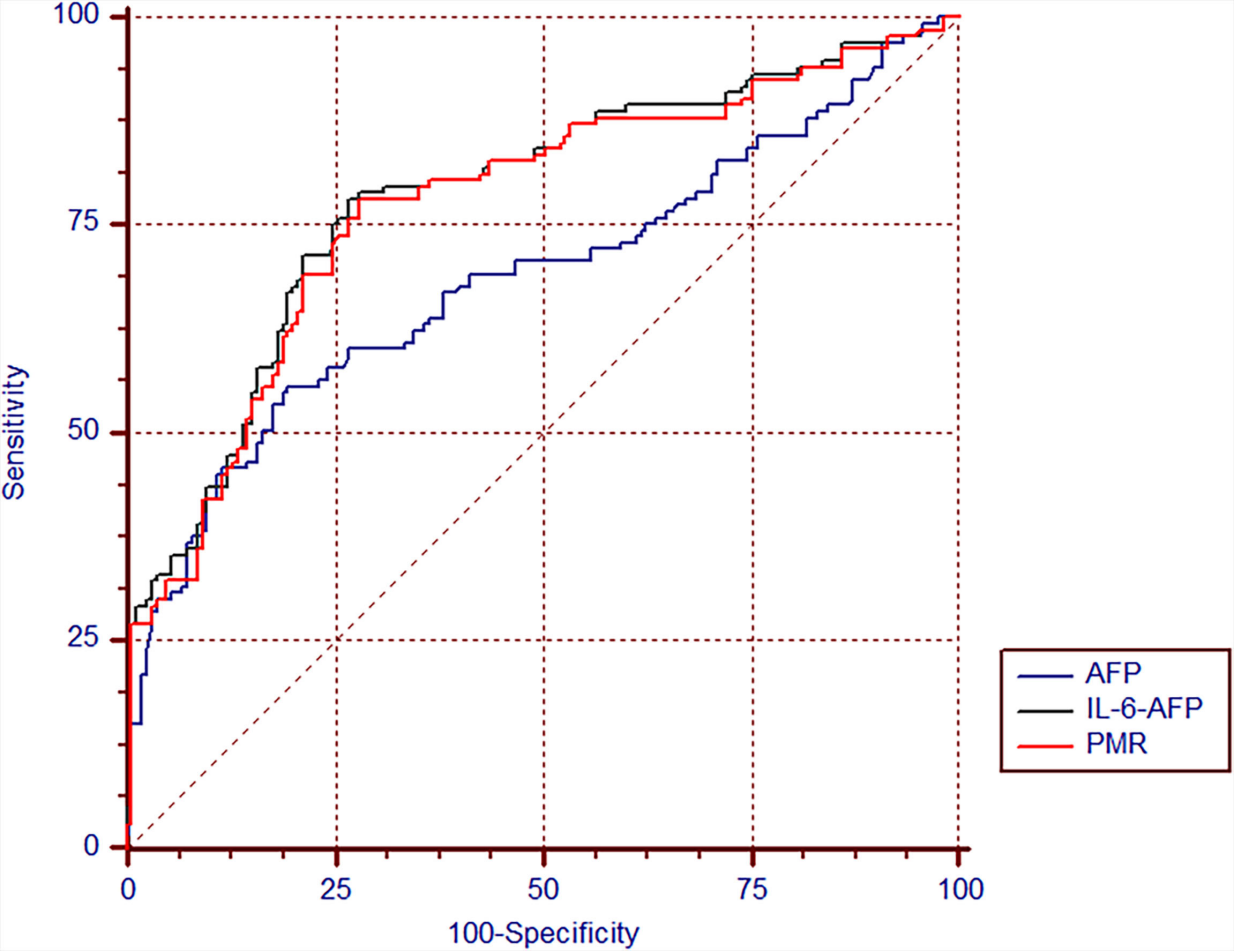
The diagnostic value of IL-6 promoter methylation levels in HBV-associated HCC. The ROC of IL-6 promoter methylation level, AFP, and the combination of both tests in discriminating HBV-associated HCC from CHB.

A receiver operating characteristic (ROC) curve was used to detect the diagnostic value of the IL-6 promoter methylation level, AFP and the combined determination. As shown in **Table 4**, the AUC of the IL-6 promoter PMR value was (AUC=0.773, 95% CI 0.721-0.819) significantly higher than that of AFP (AUC=0.686, 95% CI 0.630-0.738, P=0.027). The selected threshold was 54.91%, the sensitivity was 78.2%, and the specificity was 72.1%. The model based on binary logistic regression was then constructed to evaluate the diagnostic value of IL-6 promoter methylation level combined with AFP. The combined detection AUC was 0.784(95% CI 0.732-0.829, P=0.011), and the sensitivity was 78.2%, the specificity was 73.3%, which was notably increased compared to AFP detection alone (P=0.011) (**Figure 6**).

**Table 4:** Diagnostic values of the IL-6 methylation level and the combined determination with AFP for distinguishing HBV-associated HCC from CHB.

Parameter	sensitivity	Specificity	Youden index	AUC	95%CI
IL-6	78.2	72.1	0.503	0.773	0.721-0.819
AFP	55.6	80.6	0.362	0.686	0.630-0.738
IL-6+AFP	78.2	73.3	0.515	0.784	0.732-0.829

### Independent Risk Factors for HBV-Associated HCC Development

Multivariate logistic regression analysis was used to assess the risk factors for HBV-associated HCC. The IL-6 promoter methylation level was divided into two subgroups by 54.91% which was the best cutoff point in the above article, and AFP was divided by 20 ng/ml as the cutoff point. As shown in **Table 5**, PMR value of IL-6 promoter<54.91% (OR=10.243, 95% CI 5.455-19.233, P <0.001), AFP> 20 ng/ml (OR=4.689, 95% CI 2.568-8.563, P <0.001) and HBV-DNA positive (OR=3.754, 95% CI 1.956-7.208, P<0.001) were independent risk factors affecting the occurrence of HBV-associated HCC.

**Table 5:** Independent risk factors for development of HCC.

Variable	OR	95%CI	P value
Gender (Male)	1.840	0.914-3.704	=0.088
Age (>50yr)	0.631	0.346-1.149	=0.132
IL-6 (<54.91%)	10.243	5.455-19.233	<0.001
AFP (>20 ng/ml)	4.689	2.568-8.563	<0.001
HBV-DNA (+)	3.754	1.956-7.208	<0.001

## Discussion

Although existing studies have shown somewhat correlation between the occurrence of liver cancer and the level of IL-6, the clinical relationship between the level of IL-6 promoter methylation and HBV-associated HCC has not yet been tested (29). This is the first study to prove that the IL-6 promoter methylation level in PBMCs of HBV-associated HCC patients is reduced compared with that in PBMCs of CHB patients. The level of IL-6 mRNA in PBMCs of HBV-associated HCC patients is markedly higher than that of CHB patients and normal controls. The PMR value of the IL-6 promoter was negatively correlated with the IL-6 mRNA level. In HBV-associated HCC, the level of IL-6 promoter methylation in males was higher than that in females, in those >50 years old than in those <50 years old, in HBV-DNA positive than in HBV-DNA negative, in tumor size>3 cm than in those<=3 cms. And IL-6 promoter methylation was positively correlated with age and TBIL. Hypomethylation of the IL-6 promoter is also an independent risk factor that affects the development of liver cancer. At the same time, based on logistic regression analysis, combined detection of IL-6 promoter methylation level and AFP can improve the diagnostic ability of AFP for HBV-associated HCC.

Hypermethylation of genes in animals will silence genes, resulting in lower gene expression (30–32), while hypomethylation will reduce gene silencing and increase gene expression (33, 34). So we guessed that the hypomethylation of IL-6 increased the expression of IL-6. In many cancers, excessive activation of IL-6/JAK/STAT3 is associated with tumor cell proliferation, migration, invasion and inhibition of antitumor immune responses, and is associated with poor tumor prognosis (19). The increased expression of the IL-6 may affect the occurrence of liver cancer through some ways, but its specific process needs further experiments to verify.

**Figure 7:**
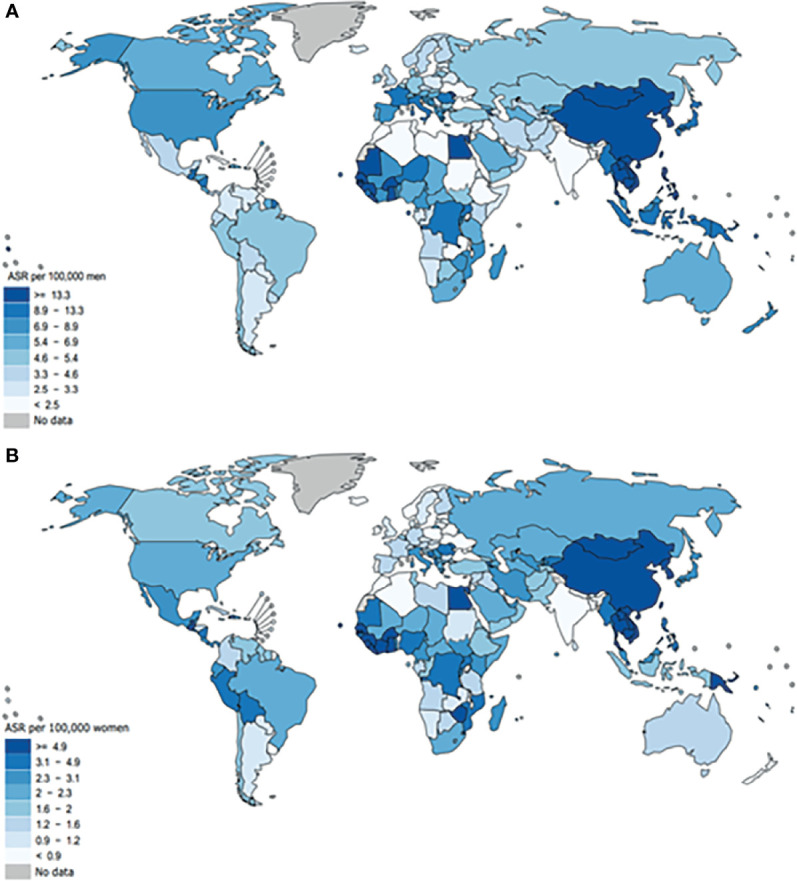
Global age-adjusted incidence rates of liver cancer, estimated for 2020. Data source: GLOBOCAN 2020. Graph production: IARC (http://gco.iarc.fr/today), World Health Organization (35). This article is protected by copyright. All rights reserved. the age-standardized rate (ASR) incidence of malein males is highest in China, Mongolia and some South Asian countries (>13.3/100,000) and then, followed by the United States and some African countries (8.9-13.3/100,000), Canada, Mexico and Oceania (6.9-8.9/100,000) and lowest in the Southeast Asia (<2.5/100,000).

Hepatocellular carcinoma (HCC), as the most common form of liver cancer, is the main cause of liver cancer diagnosis and death (1). In different areas of the world, HCC is quite infrequent. Based on the GLOBOCAN 2020, the age-standardized rate (ASR) incidence in males is highest in China, Mongolia and some South Asian countries (>13.3/100,000) and then followed by United States and some African countries (8.9-13.3/100,000), Canada, Mexico and Oceania (6.9-8.9/100,000) and lowest in the Southeast Asia (<2.5/100,000) as represented in **Figure 7** (35). The commonly used treatment methods for liver cancer are Barcelona Clinic Liver Cancer approach (36) and the new EASL Clinical Practice Guidelines (37), and the late effect will not be effective, so early detection and early treatment are necessary. In China, the main risk factor for HCC is HBV (1, 3, 4). Because early diagnosis is difficult, a variety of patients are diagnosed with HCC at an advanced stage (8). Recent studies have found that abnormal DNA methylation is related to a great number of human diseases. The DNA methylation status of free cells is similar to that of primary tumor tissues (26, 38). IL-6 synthesis and secretion are affected by inflammatory conditions (18). Meanwhile, it is related to breast cancer staging and prognosis (39), esophageal squamous cell carcinoma (40, 41), oral squamous cell carcinoma (42), epithelial ovarian cancer (43) and other tumors. Interleukin-6 (IL-6) has been confirmed to participate in the occurrence of HBV-related liver cancer by activating the STAT3 pathway (19). Studies have shown that the level of serum IL-6 after treatment can be used as a powerful predictor of the prognosis of HCC (29). In youth prehypertension, the IL-6 promoter is inhibited by DNA methylation, and IL-6 gene CpG methylation can be used as a surrogate biomarker for the diagnosis of childhood IBD (20, 21). Increased serum IL-6 downregulated PTPRO expression in HCC monocytes (44). In order to evaluate the diagnostic value of the IL-6 promoter methylation level as a noninvasive biomarker, we selected PBMCs from HCC patients as the research specimens, using a quantitative high flux methylation detection method–methylight test, which has higher sensitivity and specificity than traditional MSP technology (15, 45). In this study, we found for the first time that IL-6 promoter hypomethylation occurred in the PBMCs of patients with HCC. The IL-6 promoter methylation level of HBV-associated HCC was clearly lower than that of CHB patients. The level of IL-6 mRNA in PBMCs and CHB patients was evidently higher than that of the normal control group. The PMR values of IL-6 promoter and IL-6 mRNA levels were obviously negatively correlated. Among them, the IL-6 promoter methylation value of HBV-related HCC patients with a tumor size >3 cm was sharply higher than that of patients with <3 cm Multivariate logistic regression analysis showed that hypomethylation of the IL-6 promoter is an independent risk factor of HCC. These results indicate that the methylation level of the IL-6 promoter may be a potential biomarker for monitoring and predicting the prognosis of HBV-related liver cancer. It is worth noting that the methylation level of the IL-6 promoter is lower in normal people, and the level of mRNA is also lower. One possibility to explain the inconsistent trends is that in addition to promoter methylation, there are many other factors that can affect the transcription process, such as microRNA, long noncoding RNAs, enhancer of noncoding RNA transcription unit, histone modifications, and transcription factors, transcription factors and the nucleosome (46–50).

Early detection and diagnosis of liver cancer can help improve survival (8). At present, AFP is still the most widely accepted serum biomarker in liver cancer in monitoring and diagnosis (51). Its sensitivity (40-60%) and specificity (65-81%) are not high using the traditional boundary of 20 ng/ml, and it has limitations as a diagnostic marker for liver cancer (7, 52, 53). In this study, the methylation level of the IL-6 promoter was quantitatively evaluated, and a ROC curve was drawn to further evaluate its diagnostic value. Experimental results exhibit that the diagnosis of the IL-6 promoter methylation level is sharply better than that of AFP. The combination of serum AFP and the PMR value of the IL-6 promoter in PBMCs can further improve the diagnostic ability of AFP. With 54.91% as the cutoff point, the sensitivity of the methylation level of the IL-6 promoter alone to distinguish HCC and CHB was 78.2%, and the specificity was 72.1%. If the currently recommended clinical cut-off point (20 ng/ml) is used, the sensitivity of AFP is 55.6% and the specificity is 80.6%. In comparison, the sensitivity of the combined detection of IL-6 promoter PMR and AFP was 78.2% and the specificity was 73.3%. The combined detection of IL-6 methylation level in PBMCs and serum AFP can sharply improve the diagnostic ability of AFP. At the same time, no correlation between IL-6 promoter methylation level and AFP was observed. The above results demonstrate that the methylation level of the IL-6 promoter may become a noninvasive diagnostic marker for liver cancer independent of AFP.

This experiment has several limitations. First, long-term follow-up data before and after the development of HCC were not enrolled. Further analysis of the prognostic value of the level of IL-6 promoter methylation in HCC is not conducted, and there was no way to conduct survival analysis. Second, the sample size was relatively small, especially since all patients were selected from a single-center, which may lead to selection bias. Third, there are some technical difficulties in the detection of DNA methylation levels by methylight, such as DNA extraction and sodium bisulfite treatment. Fourth, the methylation level of the IL-6 promoter in serum was not compared with that in liver tissue. However, studies have displayed that the methylation status of cell free DNA is similar to the methylation status of the primary tumor tissue [13,14]. Finally, the super minor changes between HCC and CHB groups in PMR percentage does not mean anything to the review. It may be because of the PBMC mixture cells were used as the target sample, but not a specific immune cell subtype was used. Meanwhile, although there is a statistical difference, the chance of representing a biological difference by 3 percent change on promoter methylation level is super low. We need further experiments to confirm. In the future, a prospective cohort follow-up study of multicenter and larger-scale patients is needed to confirm our point of view.

## Conclusion

In conclusion, we discovered that the IL-6 promoter methylation level in HBV-associated HCC was significantly lower than that in CHB patients, and the IL-6 mRNA level in HBV-associated HCC was significantly higher than that of CHB patients and normal people. The level of IL-6 promoter methylation, an independent factor in the development of liver cancer, is negatively correlated with the IL-6 mRNA level. At the same time, the methylation level of the IL-6 promoter as a noninvasive biomarker is more sensitive and specific than AFP in the diagnosis of HCC. Therefore, methylation of the IL-6 promoter may become a potential biomarker to detect HCC.

## Data Availability Statement

The original contributions presented in the study are included in the article/supplementary material. Further inquiries can be directed to the corresponding author.

## Ethics Statement

The studies involving human participants were reviewed and approved by the local Ethical Committee of Qilu Hospital of Shandong University. The patients/participants provided their written informed consent to participate in this study.

## Author Contributions

JY designed the study, performed the research, analyzed the data and wrote the paper. JW and HL revised the paper. SG helped with the statistical analysis. YF contributed to the formal analysis, writing- review and editing. KW contributed to the supervision, design of the study and the critical revision of the paper. KW guaranteed the article. All authors contributed to the article and approved the submitted version.

## Funding

This work was supported by the National Natural Science Foundation of China (81970522), National Key Research and Development Program of China (No. 2021YFC2301800), Shandong University multidisciplinary research and innovation team of young scholars (2020QNQT11), China Postdoctoral Science Foundation (2020M672074), and the Young Taishan Scholars (tsqn202103169).

## Conflict of Interest

The authors declare that the research was conducted in the absence of any commercial or financial relationships that could be construed as a potential conflict of interest.

## Publisher’s Note

All claims expressed in this article are solely those of the authors and do not necessarily represent those of their affiliated organizations, or those of the publisher, the editors and the reviewers. Any product that may be evaluated in this article, or claim that may be made by its manufacturer, is not guaranteed or endorsed by the publisher.

## References

[1] McGlynn KA Petrick JL El-Serag HB. Epidemiology of Hepatocellular Carcinoma. Hepatology (2021) 73 Suppl 1:4–13. doi: 10.1002/hep.31288 PMC7577946 32319693

[2] today C. International Agency for Research on Cancer. Geneva: World Health Organization (2020). Available at: https://gco.iarc.fr/today/home.

[3] Llovet JM Kelley RK Villanueva A Singal AG Pikarsky E Roayaie S. Hepatocellular Carcinoma. Nat Rev Dis Primers (2021) 7(1):6. doi: 10.1038/s41572-020-00240-3 33479224 PMC13537433

[4] Chaturvedi VK Singh A Dubey SK Hetta HF John J Singh MP. Molecular Mechanistic Insight of Hepatitis B Virus Mediated Hepatocellular Carcinoma. Microb Pathog (2019) 128:184–94. doi: 10.1016/j.micpath.2019.01.004 30611768

[5] European Association for the Study of the Liver. Electronic Address Eee, European Association for the Study of the L. EASL Clinical Practice Guidelines: Management of Hepatocellular Carcinoma. J Hepatol (2018) 69(1):182–236. doi: 10.1016/j.jhep.2018.03.019 29628281

[6] Marrero JA Kulik LM Sirlin CB Zhu AX Finn RS Abecassis MM. Diagnosis, Staging, and Management of Hepatocellular Carcinoma: 2018 Practice Guidance by the American Association for the Study of Liver Diseases. Hepatol (2018) 68(2):723–50. doi: 10.1002/hep.29913 29624699

[7] Bejarano PA. Diagnostic and Prognostic Role of α-Fetoprotein in Hepatocellular Carcinoma: Both or Neither? Yearbook Pathol Lab Med (2008) 2008:66–7. doi: 10.1016/S1077-9108(08)70566-8

[8] Fu J Wang H. Precision Diagnosis and Treatment of Liver Cancer in China. Cancer Lett (2018) 412:283–8. doi: 10.1016/j.canlet.2017.10.008 29050983

[9] Villanueva A Longo DL. Hepatocellular Carcinoma. New Engl J Med (2019) 380(15):1450–62. doi: 10.1056/NEJMra1713263 30970190

[10] Kelley RK Greten TF. Hepatocellular Carcinoma - Origins and Outcomes. N Engl J Med (2021) 385(3):280–2. doi: 10.1056/NEJMcibr2106594 34260842

[11] Weisenberger DJ Siegmund KD Campan M Young J Long TI Faasse MA. CpG Island Methylator Phenotype Underlies Sporadic Microsatellite Instability and is Tightly Associated With BRAF Mutation in Colorectal Cancer. Nat Genet (2006) 38(7):787–93. doi: 10.1038/ng1834 16804544

[12] Kerachian MA Javadmanesh A Azghandi M Mojtabanezhad Shariatpanahi A Yassi M Shams Davodly E. Crosstalk Between DNA Methylation and Gene Expression in Colorectal Cancer, a Potential Plasma Biomarker for Tracing This Tumor. Sci Rep (2020) 10(1):2813. doi: 10.1038/s41598-020-59690-0 32071364 PMC7028731

[13] Constancio V Nunes SP Henrique R Jeronimo C. DNA Methylation-Based Testing in Liquid Biopsies as Detection and Prognostic Biomarkers for the Four Major Cancer Types. Cells (2020) 9(3):624. doi: 10.3390/cells9030624 PMC7140532 32150897

[14] Liu L Toung JM Jassowicz AF Vijayaraghavan R Kang H Zhang R. Targeted Methylation Sequencing of Plasma Cell-Free DNA for Cancer Detection and Classification. Ann Oncol (2018) 29(6):1445–53. doi: 10.1093/annonc/mdy119 PMC6005020 29635542

[15] Trinh BN Long TI Laird PW. DNA Methylation Analysis by MethyLight Technology. Methods (2001) 25(4):456–62. doi: 10.1006/meth.2001.1268 11846615

[16] Bartak BK Kalmar A Peterfia B Patai AV Galamb O Valcz G. Colorectal Adenoma and Cancer Detection Based on Altered Methylation Pattern of SFRP1, SFRP2, SDC2, and PRIMA1 in Plasma Samples. Epigenetics (2017) 12(9):751–63. doi: 10.1080/15592294.2017.1356957 PMC5739100 28753106

[17] Cindy A Eads KDD Kawakami K Saltz LB Blake C Shibata D. MethLight: A High-Throughput Assay to Measure DNA Methylation. Nucleic Acids Res (2000) 28(8):E32. doi: 10.1093/nar/28.8.e32 10734209 PMC102836

[18] Kishimoto T. IL-6: From its Discovery to Clinical Applications. Int Immunol (2010) 22(5):347–52. doi: 10.1093/intimm/dxq030 20410258

[19] Johnson DE O’Keefe RA Grandis JR. Targeting the IL-6/JAK/STAT3 Signalling Axis in Cancer. Nat Rev Clin Oncol (2018) 15(4):234–48. doi: 10.1038/nrclinonc.2018.8 PMC5858971 29405201

[20] Omar W Abdullah A Talib NA Shah ASM Rahman JA. Leucocytic DNA Methylation of Interleukin-6 Promoter Reduction in Pre-Hypertensive Young Adults. Malays J Med Sci (2019) 26(6):46–54. doi: 10.21315/mjms2019.26.6.5 31908586 PMC6939726

[21] Samarani S Dupont-Lucas C Marcil V Mack D Israel D Deslandres C. CpG Methylation in TGFbeta1 and IL-6 Genes as Surrogate Biomarkers for Diagnosis of IBD in Children. Inflammation Bowel Dis (2020) 26(10):1572–8. doi: 10.1093/ibd/izaa074 32407484

[22] Yuan K Lei Y Chen HN Chen Y Zhang T Li K. HBV-Induced ROS Accumulation Promotes Hepatocarcinogenesis Through Snail-Mediated Epigenetic Silencing of SOCS3. Cell Death Differ (2016) 23(4):616–27. doi: 10.1038/cdd.2015.129 PMC4986634 26794444

[23] Terrault NA Lok ASF McMahon BJ Chang KM Hwang JP Jonas MM. Update on Prevention, Diagnosis, and Treatment of Chronic Hepatitis B: AASLD 2018 Hepatitis B Guidance. Hepatol (2018) 67(4):1560–99. doi: 10.1002/hep.29800 PMC5975958 29405329

[24] Xiang L Chen LM Zhai YJ Sun WJ Yang JR Fan YC. Hypermethylation of Secreted Frizzled Related Protein 2 Gene Promoter Serves as a Noninvasive Biomarker for HBV-Associated Hepatocellular Carcinoma. Life Sci (2021) 270:119061. doi: 10.1016/j.lfs.2021.119061 33454364

[25] Mohammadpanah M Heidari MM Khatami M Hadadzadeh M. Relationship of Hypomethylation CpG Islands in Interleukin-6 Gene Promoter With IL-6 mRNA Levels in Patients With Coronary Atherosclerosis. J Cardiovasc Thorac Res (2020) 12(3):214–21. doi: 10.34172/jcvtr.2020.37 PMC7581844 33123328

[26] Gao S Sun FK Fan YC Shi CH Zhang ZH Wang LY. Aberrant GSTP1 Promoter Methylation Predicts Short-Term Prognosis in Acute-on-Chronic Hepatitis B Liver Failure. Aliment Pharmacol Ther (2015) 42(3):319–29. doi: 10.1111/apt.13271 26040771

[27] European Association For The Study Of The L European Organisation For R Treatment Of C. EASL-EORTC Clinical Practice Guidelines: Management of Hepatocellular Carcinoma. J Hepatol (2012) 56(4):908–43. doi: 10.1016/j.jhep.2011.12.001 22424438

[28] Park EJ Lee JH Yu GY He G Ali SR Holzer RG. Dietary and Genetic Obesity Promote Liver Inflammation and Tumorigenesis by Enhancing IL-6 and TNF Expression. Cell (2010) 140(2):197–208. doi: 10.1016/j.cell.2009.12.052 20141834 PMC2836922

[29] Wu Y Fan W Xue M Zhong B Zhang S Wang Y. Postintervention Interleukin-6 (IL-6) Level, Rather Than the Pretreatment or Dynamic Changes of IL-6, as an Early Practical Marker of Tumor Response in Hepatocellular Carcinoma Treated With Transarterial Chemoembolization. Oncologist (2019) 24(12):e1489–e95. doi: 10.1634/theoncologist.2018-0669 PMC6975952 31249138

[30] Lao TD Nguyen TN Le TAH. Promoter Hypermethylation of Tumor Suppressor Genes Located on Short Arm of the Chromosome 3 as Potential Biomarker for the Diagnosis of Nasopharyngeal Carcinoma. Diagn (Basel) (2021) 11(8):1404. doi: 10.3390/diagnostics11081404 PMC8391633 34441339

[31] Zardo G. The Role of H3K4 Trimethylation in CpG Islands Hypermethylation in Cancer. Biomolecules (2021) 11(2):143. doi: 10.3390/biom11020143 33499170 PMC7912453

[32] Zhao J Cao H Zhang W Fan Y Shi S Wang R. SOX14 Hypermethylation as a Tumour Biomarker in Cervical Cancer. BMC Cancer (2021) 21(1):675. doi: 10.1186/s12885-021-08406-2 34098886 PMC8185922

[33] Duan X Huang Y Chen X Wang W Chen J Li J. Moderate DNA Hypomethylation Suppresses Intestinal Tumorigenesis by Promoting Caspase-3 Expression and Apoptosis. Oncogenesis (2021) 10(5):38. doi: 10.1038/s41389-021-00328-9 33947834 PMC8096944

[34] Ma Q Caillier SJ Muzic S Wilson MR Henry RG Cree BAC. Specific Hypomethylation Programs Underpin B Cell Activation in Early Multiple Sclerosis. Proc Natl Acad Sci USA (2021) 118(51):e2111920118. doi: 10.1073/pnas.2111920118 34911760 PMC8713784

[35] Rumgay H Ferlay J de Martel C Georges D Ibrahim AS Zheng R. Global, Regional and National Burden of Primary Liver Cancer by Subtype. Eur J Cancer (2022) 161:108–18. doi: 10.1016/j.ejca.2021.11.023 34942552

[36] Munoz-Martinez S Iserte G Sanduzzi-Zamparelli M Llarch N Reig M. Current Pharmacological Treatment of Hepatocellular Carcinoma. Curr Opin Pharmacol (2021) 60:141–8. doi: 10.1016/j.coph.2021.07.009 34418875

[37] Bruix J Chan SL Galle PR Rimassa L Sangro B. Systemic Treatment of Hepatocellular Carcinoma: An EASL Position Paper. J Hepatol (2021) 75(4):960–74. doi: 10.1016/j.jhep.2021.07.004 34256065

[38] Al-Moghrabi N Al-Qasem AJ Aboussekhra A. Methylation-Related Mutations in the BRCA1 Promoter in Peripheral Blood Cells From Cancer-Free Women. Int J Oncol (2011) 39(1):129–35. doi: 10.3892/ijo.2011.1021 21537840

[39] Sansone P Storci G Tavolari S Guarnieri T Giovannini C Taffurelli M. IL-6 Triggers Malignant Features in Mammospheres From Human Ductal Breast Carcinoma and Normal Mammary Gland. J Clin Invest (2007) 117(12):3988–4002. doi: 10.1172/JCI32533 18060036 PMC2096439

[40] Zheng Y Li Y Tang B Zhao Q Wang D Liu Y. IL-6-Induced CD39 Expression on Tumor-Infiltrating NK Cells Predicts Poor Prognosis in Esophageal Squamous Cell Carcinoma. Cancer Immunol Immunother (2020) 69(11):2371–80. doi: 10.1007/s00262-020-02629-1 PMC11027717 32524362

[41] Wu J Gao FX Wang C Qin M Han F Xu T. IL-6 and IL-8 Secreted by Tumour Cells Impair the Function of NK Cells *via* the STAT3 Pathway in Oesophageal Squamous Cell Carcinoma. J Exp Clin Cancer Res (2019) 38(1):321. doi: 10.1186/s13046-019-1310-0 31324197 PMC6642486

[42] Marton IJ Horvath J Labiscsak P Markus B Dezso B Szabo A. Salivary IL-6 mRNA is a Robust Biomarker in Oral Squamous Cell Carcinoma. J Clin Med (2019) 8(11):1958. doi: 10.3390/jcm8111958 PMC6912409 31766212

[43] De Marco1 M Festa M Raffone A Sandullo L Rosati A Reppucci F. Different Mechanisms Underlie IL-6 Release in Chemosensitive and Chemoresistant Ovarian Carcinoma Cells. Am J Cancer Res (2020) 10(8):2596–602. eCollection 2020. PMC7471370 32905525

[44] Zhang W Liu Y Yan Z Yang H Sun W Yao Y. IL-6 Promotes PD-L1 Expression in Monocytes and Macrophages by Decreasing Protein Tyrosine Phosphatase Receptor Type O Expression in Human Hepatocellular Carcinoma. J Immunother Cancer (2020) 8(1):e000285. doi: 10.1136/jitc-2019-000285 32581055 PMC7319788

[45] Olkhov-Mitsel E Zdravic D Kron K van der Kwast T Fleshner N Bapat B. Novel Multiplex MethyLight Protocol for Detection of DNA Methylation in Patient Tissues and Bodily Fluids. Sci Rep (2014) 4:4432. doi: 10.1038/srep04432 24651255 PMC3961737

[46] Bannister AJ Kouzarides T. Regulation of Chromatin by Histone Modifications. Cell Res (2011) 21(3):381–95. doi: 10.1038/cr.2011.22 PMC3193420 21321607

[47] Kozomara A Birgaoanu M Griffiths-Jones S. Mirbase: From microRNA Sequences to Function. Nucleic Acids Res (2019) 47(D1):D155–D62. doi: 10.1093/nar/gky1141 PMC6323917 30423142

[48] Li W Notani D Rosenfeld MG. Enhancers as non-Coding RNA Transcription Units: Recent Insights and Future Perspectives. Nat Rev Genet (2016) 17(4):207–23. doi: 10.1038/nrg.2016.4 26948815

[49] West JA Davis CP Sunwoo H Simon MD Sadreyev RI Wang PI. The Long Noncoding RNAs NEAT1 and MALAT1 Bind Active Chromatin Sites. Mol Cell (2014) 55(5):791–802. doi: 10.1016/j.molcel.2014.07.012 25155612 PMC4428586

[50] Zhu F Farnung L Kaasinen E Sahu B Yin Y Wei B. The Interaction Landscape Between Transcription Factors and the Nucleosome. Nature (2018) 562(7725):76–81. doi: 10.1038/s41586-018-0549-5 30250250 PMC6173309

[51] Galle PR Foerster F Kudo M Chan SL Llovet JM Qin S. Biology and Significance of Alpha-Fetoprotein in Hepatocellular Carcinoma. Liver Int (2019) 39(12):2214–29. doi: 10.1111/liv.14223 31436873

[52] Lok AS Sterling RK Everhart JE Wright EC Hoefs JC Di Bisceglie AM. Des-Gamma-Carboxy Prothrombin and Alpha-Fetoprotein as Biomarkers for the Early Detection of Hepatocellular Carcinoma. Gastroenterology (2010) 138(2):493–502. doi: 10.1053/j.gastro.2009.10.031 19852963 PMC2819612

[53] Beudeker BJB Boonstra A. Circulating Biomarkers for Early Detection of Hepatocellular Carcinoma. Therap Adv Gastroenterol (2020) 13:1756284820931734. doi: 10.1177/1756284820931734 PMC7325534 32647536

